# Effects of the COVID-19 pandemic on public bus occupancy and real-world tailpipe emissions of gaseous pollutants per passenger kilometer traveled

**DOI:** 10.1186/s42834-022-00146-7

**Published:** 2022-08-09

**Authors:** Narayan Babu Dhital, Lin-Chi Wang, Hsi-Hsien Yang, Che-Hsuan Lee, Wei-Hung Shih, Cheng-Shiu Wu

**Affiliations:** 1 Department of Environmental Engineering and Management, Chaoyang University of Technology, Taichung City, 413310 Taiwan; 2 Department of Environmental Science, Patan Multiple Campus, Tribhuvan University, Patandhoka, 44700 Nepal; 3 Department of Environmental Engineering, Chung Yuan Christian University, Taoyuan City, 320314 Taiwan; 4 Center for Environmental Risk Management, Chung Yuan Christian University, Taoyuan City, 320314 Taiwan

**Keywords:** Break-even passenger occupancy, Emission factor, Gaseous pollutant, Occupancy rate, On-road emission, Public transportation, Real-world emission

## Abstract

Public buses typically have less emission per passenger kilometer traveled (PKT) than private cars and motorcycles, and the emission benefit of public buses increases with ridership. However, the drop in public bus usage during the novel coronavirus (COVID-19) pandemic could lead to an increase in air pollutant emissions per PKT, making the emission benefits of public buses questionable. This study investigated the effects of the COVID-19 pandemic on public bus occupancy rates in Taichung City, Taiwan, and also compared real-world emissions per PKT of carbon monoxide (CO), total hydrocarbons (THC), nitric oxide (NO), and carbon dioxide (CO_2_) of a public bus before and during the pandemic. Mean bus occupancy rates were 11–25% on different bus routes before the pandemic, indicating that only a fourth or less of the bus passenger capacity was utilized. During the pandemic, mean bus occupancy rates dropped to 4–15%. Moreover, the public bus was less polluting based on CO and THC emissions than the car and motorcycle, even at the low passenger occupancy rates observed during the pandemic. However, NO and CO_2_ emissions per PKT of the bus were remarkably higher during the pandemic than those of the car and motorcycle. Furthermore, we estimated the break-even passenger occupancy rate for buses as 15%, which was the minimum threshold occupancy rate below which the buses would be more polluting than cars and motorcycles in terms of CO, THC, and CO_2_ emissions per PKT. Our findings will help transport management authorities and policymakers to optimize bus route designs and frequencies and implement anti-pandemic measures to maximize the environmental benefits of the public bus transit systems.

**Supplementary Information:**

The online version contains supplementary material available at 10.1186/s42834-022-00146-7.

## Introduction

Public transport is more desirable than private modes of transport and is often subsidized by governments in cities around the world [1]. Public transport systems, such as bus transit, are claimed to offer a multitude of environmental benefits. One of such benefits is a relatively lower emission intensity, measured as emissions per passenger kilometer traveled (PKT), of public than private transport modes. However, the emission benefit from public bus transit strongly depends on ridership (number of passengers on each trip) [2, 3]. Although the total emission of a bus can be expected to increase with increasing passenger load (ridership), the emissions per PKT are often lower at higher passenger loads because the total emission would be divided among more passengers [3, 4]. Therefore, keeping everything else constant, the amount of air pollutants emitted and fuel consumption (FC) of public buses per PKT increase with decreasing ridership.

With the onset of the novel coronavirus disease (COVID-19), several countries around the globe have been imposing curtailments on outdoor movement and activities of people in an effort to keep the pandemic from further spreading. The restrictions include lockdowns and the requirement to maintain social distancing. Those restrictions have severely affected mobility and the use of public transportation in many countries [5–9]. Passengers are likely to get the COVID-19 infection in public buses as a recent study showed that the virus may remain suspended in the air inside buses [10]. Due to the fear of getting infected during public bus rides, people would shift their preference from public to private modes of transportation [2].

Many studies have shown that the pandemic and the subsequent implementation of anti-pandemic measures have dramatically changed travel behavior and mode choices [2], and the transit bus ridership has significantly dropped in several big cities [5, 7, 8]. For example, Sahraei et al. [9] showed that the public transport usage in twelve different countries in Europe and America decreased up to 90% during the pandemic. This massive drop in bus ridership leads to the increase in air pollutant emissions per passenger per kilometer of distance traveled, making the emission benefits of public buses over private modes of transportation uncertain [2]. Therefore, the positive environmental effects of public buses over private modes of transportation may be overturned due to the pandemic. For instance, a study suggested that public buses were more polluting than private cars based on emissions per PKT when the passenger ridership dropped by more than 40% during the COVID-19 pandemic compared to the pre-pandemic ridership [2]. In contrast, another study suggested that with the effective implementation of anti-pandemic measures, such as proper use of face masks, sanitization, and social distancing, it might be possible to maintain sufficient public bus ridership [11]. Therefore, it is necessary to evaluate the environmental impacts, especially emissions of air pollutants, of public bus transit systems under the changing scenario due to the COVID-19 pandemic based on pollutant emissions per PKT of buses.

Bus transit is a common and often subsidized means of public transportation in many countries and cities [1, 12], including Taiwan. In Taiwan, Taichung is the second-largest city after Taipei. Of the total travel trips made over the whole country in 2020, 11.5% share was of Taichung City alone [13]. The travel mode is highly dominated by private transportation, with the modal share of public, private, and non-motorized transport (cycling and walking) being approximately 9, 81, and 10%, respectively, in 2020 [13]. However, of all trips made by public transport in the city, 42.4% were made by public buses only [13], which indicates that public bus transit is the most common mode of public transportation in Taichung City, with 255 public bus routes in operation as of 2021.

Despite the global outbreak of the COVID-19 pandemic from the beginning of 2020, there was never a complete lockdown in Taichung City, Taiwan. Therefore, the pandemic did not affect the public bus operation schedule and frequency in the city. Nonetheless, it might have still affected the travel mode preference of people and hence the public bus ridership and occupancy. The historic experience of the 2003-SARS epidemic in Taiwan showed that the fear of getting infected during the disease outbreak period may cause an immediate drop in public transport ridership following the reports of new cases of the disease [14]. However, the effects of the ongoing COVID-19 pandemic on public bus usage and tailpipe emissions per PKT are yet to be investigated in Taiwan.

Recently, with the widespread vaccination programs and periodic control over the pandemic, the restrictions have been loosened. Consequently, the public bus ridership has started to increase slowly. However, due to the persistence of the pandemic, studies have indicated that the public transit ridership may not reach the pre-pandemic levels any time soon [5, 8, 12]. The requirement of social distancing and public fear of disease transmission can be expected to persist longer, and it might take a long period—several months to years in the future—for the public transportation ridership to reach the normal or pre-pandemic level. This gradual process of change provides opportunities to improve the urban transportation system [8]. Therefore, it may be necessary to re-examine the environmental aspects and overall merit of public bus transit systems that would help identify improvement opportunities.

Several prior studies have analyzed the effect of the COVID-19 pandemic and subsequent restrictions, such as lockdowns, on ambient air quality [9, 15–19]. Most of those studies showed that less vehicular traffic and the decrease in other emission activities during the pandemic improved ambient air quality. However, very few prior studies have analyzed the effect of the pandemic on the occupancy rates and tailpipe emissions per PKT of the public bus transit systems [2]. Even those studies are based on the estimated emission factors rather than the real-world measured emission factors.

In this context, we investigated the effects of the COVID-19 pandemic on public bus occupancy rates and tailpipe emissions per PKT of carbon monoxide (CO), total hydrocarbons (THC), nitric oxide (NO), carbon dioxide (CO_2_), and FC of the public buses in Taichung City, Taiwan, based on the real-world emission measurement and ridership data. The study also compared the emission factors per PKT of public buses before and during the pandemic with those of private modes of transportation (car and motorcycle) and calculated the break-even passenger occupancy rates for buses—the minimum threshold occupancy rate below which the buses would be more polluting than cars and motorcycles in terms of emissions per PKT. This is the first study that compared real-world public bus emissions per PKT before and during the COVID-19 pandemic. The results of this study will help policymakers and transport management authorities make better decisions and optimize the public bus route designs, bus frequencies, social distancing measures, etc., in the changing scenario caused by the COVID-19 pandemic.

## Methods

### Study site and the selection of transportation modes

This study was conducted in Taichung City, Taiwan. Mass transit rail, high-speed rail, taxicabs, and buses are some of the public transport options available in Taichung City. However, based on the modal share statistics, buses are a major means of public transportation [13]. Likewise, among the private transportation modes, motorcycles and cars are the most common modes in the city [13]. Therefore, in this study, we selected city buses to represent public transport and motorcycles and cars to represent private transport modes for the comparison of emissions per PKT.

### Sample bus routes and ridership data

Four public bus routes operating in Taichung City (route-59, 131, 132, and 133), Taiwan, were selected for analyzing the effect of the COVID-19 pandemic on public bus occupancy. The route-59 buses are operated by United Bus Company while the remaining three route buses are operated by Taichung Bus Company. These routes connect suburban areas with downtown Taichung City and go through different urban land-use types. All of these routes go via one of the two major train stations in the city (Taichung Railway Station and Taichung High-Speed Railway Station), thus carrying connecting passengers to the train stations. The route lengths were 23.0 km (route-59), 25.7 km (route-131), 20.6 km (route-132), and 16.3 km (route-133). A map of the sample routes is presented in the Supplementary Material (Fig. S1).

The number of passengers boarding and alighting at each bus stop for all bus trips was obtained from the bus company for the period of 25 months, from September 2019 to September 2021, for each sample route. Then, the ridership data were aggregated on a monthly basis. The data included approximately 138 thousand complete bus trips and a total of more than 4 million ridership. The 25-month data represented both the pre-pandemic and during-pandemic scenarios. The first COVID-19 case was reported in Taiwan in January 2020 [20]. Therefore, this study defines the pre-pandemic period as the period before January 2020 (that is, September to December 2019) and the during-pandemic period as the period from January 2020 to September 2021. Until January 20, 2020, there were no restrictions related to the COVID-19 pandemic in Taiwan. However, from January 21, 2020, Taiwan imposed a Level-1 alert (basic control measures) to control the pandemic on the island. Taiwan imposed the most restrictive alert (Level-3) from May 19 to July 26, 2021, during which the island observed the highest number of daily COVID-19 cases. The timeline of the pandemic-related alerts and the definitions of the alert levels are provided in the Supplementary Materials (Table S1).

### Calculation of PKT and bus occupancy

Monthly PKT was calculated using the monthly aggregated ridership data for each of the analyzed bus routes over the study period. For this, the bus route was segmented using all bus stops along the route, and the length of each route segment was calculated. It should be noted that the number and location of the bus stops on a route kept changing slightly during the sample period. Therefore, the route segmentation was done based on the bus stop locations and numbers effective during a particular month. After that, using the monthly ridership data, the total number of passengers taking the bus ride on each segment was calculated for each month. Then, PKT for each route segment was obtained by multiplying the route segment length by the total number of passengers taking the bus ride in a month on the same route segment. Finally, the total PKT for the whole route for a month was obtained by summing the PKT for individual route segments (Eq. (1)).

1 $${PKT}_{actual}=\sum_{i=1}^n{p}_i\bullet {l}_i$$

where *PKT*_*actual*_ is the actual monthly total PKT on a bus route, *p*_*i*_ is the total number of passengers taking the bus ride on the *i*^*th*^ segment of the route in a month, *l*_*i*_ (km) is the length of the route segment *i*, and *n* is the total number of route segments on the bus route. The above equation gives the monthly total PKT on a bus route in terms of person-km.

The maximum theoretical PKT, expressed in terms of person-km, which served as the reference value for comparison with the actual PKT, for each route on a month was also calculated by assuming that the bus was occupied fully for the whole route length during all bus trips on the route (Eq. (2)).

2 $${PKT}_{ref}={p}_{max}\bullet L\bullet N$$

where *PKT*_*ref*_ is the maximum theoretical PKT, *p*_*max*_ is the maximum passenger number capacity of a bus when occupied fully (sum of the seated and standing passenger capacities of a bus obtained from the vehicle registration record), *L* (km) is the total route length, and *N* is the total number of full bus trips in a month. The total number of bus trips (*N*) in a particular month for a route was calculated using the bus schedule effective during that month (Table S2). The maximum passenger capacity per bus (*p*_*max*_) obtained from the vehicle registration record was 65 (route-59), 54 (route-131), 51 (route-132), and 54 (route-133).

Researchers have used vehicle occupancy to evaluate greenhouse gas emissions and energy intensities of passenger transport [21]. The direct comparison of passenger counts between different bus routes or transport modes can be misleading because of the difference in seat capacities of different vehicles. Therefore, in the present study, a standardized metric called occupancy rate, defined as the ratio of actual PKT to the maximum theoretical PKT (or reference PKT) expressed in percentage, was used as an indicator of the intensity of public bus usage. The monthly average bus occupancy rate was calculated using Eq. (3).

3 $$Bus\ occupancy\ rate=\frac{PKT_{actual}}{PKT_{ref}}\bullet 100$$

In the above equation, a 100% occupancy rate means that the bus is fully occupied to the maximum capacity for the whole route length in all bus trips made in a month. Using the above method, the monthly average bus occupancy rates on each sample route were calculated for the period of 25 months, from September 2019 to September 2021.

Additionally, the average distance traveled by a passenger on a bus ride was also calculated (Eq. (4)).

4 $$Trip\ length\ per\ bus\ ride=\frac{PKT_{actual}}{Ridership}$$

where *PKT*_*actual*_ is the monthly total PKT on a particular bus route and *ridership* is the monthly total number of passengers taking the bus ride on the same bus route. Eq. (4) provides the average length of the trip (km) per bus ride made by a passenger for a month.

### Measurement of emission factors for bus and car

A diesel bus operating on route-59 and a gasoline car were selected for real-world emission measurement. Detailed specifications of the test vehicles are presented in Table 1. The bus was equipped with a diesel oxidation catalyst and a selective catalytic reduction device and belonged to the Taiwan Phase-5 emission certification level (equivalent to Euro V). Likewise, the car was equipped with a three-way catalytic converter and belonged to the Taiwan Phase-4 emission certification level. All emission tests for the bus and car were conducted on route-59 (Fig. S1). Four on-road emission tests (two during rush hours and two during the lean hours) were conducted for each vehicle type. The total distance driven for the emission tests was more than 180 km.

**Table 1:** Test vehicle characteristics and the sample size

Parameters	Bus	Car
Make	King Long	Toyota
Model year	2013	2008
Engine displacement volume (cm^3^)	6692	1497
Tailpipe emission control	DPF ^a^, SCR ^b^	TWC ^c^
Emission certification	Phase-5 ^d^	Phase-4 ^e^
Accumulated mileage (km)	750,550	228,200
Fuel	Diesel	Gasoline
Number of on-road emission tests	4	4
Total distance driven for on-road emission tests (km)	92.3	91.5
Number of valid data points ^f^	25,320	15,835

^a^ Diesel particulate filter

^b^ Selective catalytic reduction

^c^ Three-way catalytic converter

^d^ Implemented in 2012 in Taiwan, the emission limits are 1.5, 0.46, 2.0, and 0.02 g kWh^− 1^ for CO, THC, NO_*x*_, and PM, respectively

^e^ Implemented in 2008 in Taiwan, the emission limits are 2.11, 0.045, and 0.07 g km^− 1^ for CO, THC, and NO_*x*_, respectively

^f^ The data recording frequency of the components of the onboard emission measurement system was 1 Hz

An onboard emission measurement system consisting of a gaseous analyzer (Horiba Mexa-584 L, Horiba, Japan), an exhaust flowmeter (EFM), auxiliary sensors (exhaust temperature, ambient temperature, pressure, and humidity), a global positioning system (VBox Sport, Racelogic, Buckingham, UK), and a power supply system was used for the real-world measurement of tailpipe emissions of gaseous pollutants. All data were recorded at the frequency of 1 Hz. The weight of the onboard sampling system was approximately 45 kg. The Horiba analyzer simultaneously measures real-time concentrations of CO, THC, CO_2_, NO, and O_2_. The exhaust flow rate for the bus was measured using a pitot tube-based EFM. However, for the car, the exhaust flow rate was calculated using the intake air mass flow rate obtained from the onboard diagnostics of the car and the air-fuel ratio obtained from the Horiba gas analyzer. The detailed method of the flow calculation is described elsewhere [22]. Furthermore, the Horiba gas analyzer removes moisture from the exhaust sample before measuring the gas concentrations (measurement on a dry basis). Therefore, a dry-to-wet correction was applied to the second-by-second gas concentration data obtained from the analyzer before the calculation of emission factors [22, 23]. The detailed method of dry-to-wet correction has been described in the authors’ prior study [22]. Moisture correction factors ranged from 0.86 ± 0.00 (mean ± standard deviation, SD) to 0.96 ± 0.02 for different real-world emission test samples with data shown in the Supplementary Material (Table S3). Second-by-second exhaust gas concentrations (measured on a dry basis) were multiplied by the mean moisture correction factor obtained for the test before further analyses.

The average full-route distance-specific emission factors were calculated for the bus and car in terms of g km^− 1^. These emission factors were further used to calculate the emissions per PKT (that is, g person^− 1^ km^− 1^), as explained in the following section. It should be noted that the present study has the limitation of ignoring the effect of passenger load on real-world emission factors. However, the sampled bus route (route-59) had very low bus occupancy rates (< 14%), even before the COVID-19 pandemic (see results). Therefore, the effect of passenger load on the distance-specific emission factors would have been negligible. Results from a prior study have also suggested that the effect of passenger load (500 - > 2000 kg) on bus emissions would be negligible within a particular speed bin, especially at speeds less than 30 km h^− 1^ [24].

### Estimation of emission factors for motorcycles

We compiled the motorcycle emission factors from prior studies conducted in Taiwan for various model year motorcycles belonging to different emission certification levels in Taiwan. The emission factors were available for Phase-3, Phase-4 [25, 26], Phase-5 [26–28], and Phase-7 motorcycles [22] from the literature and are summarized in the Supplementary Material (Table S4). Then, the emission factors for each pollutant obtained from the literature were averaged for different model year classes (Table S5). Finally, the average emission factors for different model year classes were weighted by the 2021 motorcycle fleet composition (% of motorcycles belonging to different model year classes) data [29] to obtain the fleet-average emission factors for the motorcycle fleet of Taiwan (Table S5). The fleet-average emission factors of pollutants for motorcycles were used for further analysis and comparisons.

### Calculation of emissions per PKT

Emissions per PKT of a pollutant can be defined as the grams of the pollutant emitted per kilometer per passenger. It can be calculated using Eq. (5).

5 $${EF}_{PKT}=\frac{EF_d\bullet L}{PKT_{actual}}$$

where *EF*_*PKT*_ (g PKT^− 1^) is the actual emission per PKT, *EF*_*d*_ (g km^− 1^) is the real-world distance-specific emission factor of the bus, *L* (km) is the total length of the route, and *PKT*_*actual*_ is the actual PKT calculated following the method explained in the previous sections (Eq. (1)).

Moreover, the reference emissions per PKT were also calculated using the maximum theoretical PKT (Eq. (6)).

6 $${EF}_{PKT, ref}=\frac{EF_d\bullet L}{PKT_{ref}}$$

where, *EF*_*PKT,ref*_ (g PKT^− 1^) is the reference emission factor per PKT of the bus, *EF*_*d*_ is the real-world distance-specific emission factor (g km^− 1^), *L* (km) is the total length of the route, and *PKT*_*ref*_ is the maximum theoretical PKT calculated using Eq. (2).

It should be noted that the actual emissions per PKT would be equal to the reference emissions per PKT when the bus occupancy rate is 100%. However, when the bus occupancy rate is below 100%, the actual emissions per PKT would be more than the reference emissions per PKT. Therefore, in the present study, the reference emission factors per PKT have been calculated and used as the yardstick of comparison. Moreover, as indicated in the previous section, the effect of passenger load on the mass emission factors for the bus were not considered in the present study. To some extent, this might have underestimated the reference emissions per PKT of the bus.

Additionally, the actual emissions per PKT were also calculated for the car (based on measured real-world emission data) and motorcycle (based on emission data compiled from the relevant literature) using Eq. (5). A similar prior study assumed the passenger number for cars to be two [2]. In the present study, the passenger numbers were assumed to be two for the car, and one for the motorcycle. Although passenger cars and motorcycles could carry more passengers than assumed, it was considered that these private modes of transportation are not meant for ride-sharing and rarely carry the passengers to the maximum capacity. Finally, the actual emissions per PKT of the bus (calculated with the passenger ridership data under pre-pandemic and during-pandemic scenarios) and those of the car and motorcycles were compared. The study design has also been summarized schematically in the Supplementary Material (Fig. S2).

### Calculation of break-even occupancy rates

Public buses can be less or more polluting in terms of emissions per PKT than private cars and motorcycles, depending on the occupancy levels and pollutant type. Break-even passenger load has been used by researchers to evaluate emissions per PKT of public buses with those of private modes of transportation [30]. In the present study, we estimated the break-even occupancy rate defined as the minimum occupancy rate required for the bus so that the bus would be less polluting in terms of emissions per PKT than both the car and motorcycles. Equation (7) was used to estimate the break-even occupancy rate in percentage.

7 $$Break- even\ occupancy=\frac{EF_{d, bus}\bullet L}{EF_{PKT,\mathit{\min}}\bullet {PKT}_{ref}}\bullet 100$$

where, *EF*_*d,bus*_ (g km^− 1^) is the distance-specific emission factor of a pollutant for the bus, *L* (km) is the total route length, *EF*_*PKT,min*_ (g PKT^− 1^) is the lowest emission factor of the pollutant among all private transport modes being evaluated (this is because if the bus has to be less emitting than private vehicles, then the lowest emission factor per PKT of private vehicles should be the upper threshold for buses), and *PKT*_*ref*_ is the reference PKT of a trip (product of passenger capacity and route length).

## Results and discussion

### Public bus ridership and occupancy rate

**Fig. 1:**
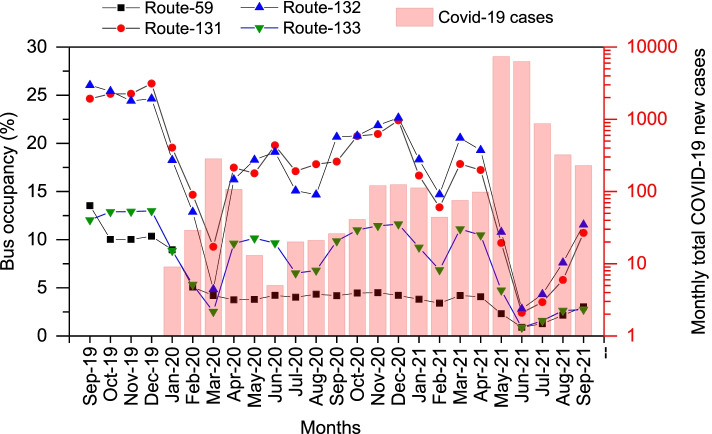
Monthly average public bus occupancy rates and monthly total COVID-19 new cases in Taiwan

Figure 1 presents time-series plots of public bus occupancy rates on routes-59, 131, 132, and 133 from September 2019 to September 2021. It also shows the monthly total COVID-19 new cases in Taiwan [20]. Irrespective of the months, the occupancy rates were similar on route-131 and 132, which were often higher than those on routes-59 and 133. The occupancy rates before the pandemic were higher than those after the pandemic on all analyzed routes. With the onset of the global COVID-19 pandemic, the occupancy rates decreased on all analyzed routes and reached the first minimum in March 2020 with the worsening scenario of the pandemic in Taiwan. On route-59, the bus occupancy rate decreased from 13.5% in September 2019 to as low as 4.2% during March 2020. Likewise, on routes-131 and 132, the pre-pandemic occupancy rates were between 24.4 and 26.2%, while those during March 2020 were as low as 9.3% (route-131) and 4.8% (route-132). The bus occupancy rates on route-133 dropped from approximately 13% during the pre-pandemic period to a minimum of 2.5% during March 2020. Therefore, the bus occupancy rates dropped to the first minimum during March 2020 on the test routes, when the first wave of the pandemic was observed in Taiwan. The pandemic remained relatively under control in Taiwan from April 2020, due to which the bus occupancy rates increased on route-131, 132, and 133, though the rates were still much less than the pre-pandemic levels. However, occupancy rates did not increase on route-59, even after March 2020, and it continued to drop below 4% until May 2020 and then remained approximately constant until April 2021. These data show that people might have reduced outdoor trips or have shifted to private modes of transportation to avoid crowds on public buses. The routes-131, 132, and 133 connect a couple of universities with downtown Taichung. Therefore, the small troughs in the time-series occupancy rates on those routes in July 2020 and February 2021 were likely due to the summer and winter breaks, respectively, at the universities, leading to the drop in the number of students taking bus rides.

The second wave of the COVID-19 pandemic was more severe in Taiwan and was observed during May–June 2021 (Fig. 1), due to which the country implemented level-2 or 3 alerts from May 11, 2021 (Table S1). This resulted in a sharp drop in public bus occupancy rates on all test routes (Fig. 1). The lowest bus occupancy rates were observed during June 2021 on all four routes. The occupancy rates during June 2021 were 0.9% (route-59), 2.4% (route-131), 2.8% (route-132), and 0.9% (route-133). After July 2021, the pandemic was again under control and the government withdrew some of the restrictions on outdoor activities and gatherings in Taiwan. Then the bus occupancy started increasing on all four routes again.

In summary, these results indicate that the public buses were largely underused in Taichung City even before the pandemic. Moreover, the analysis of public bus occupancy rates during the 2-year period, representing the pre-pandemic and during-pandemic scenarios, showed that the COVID-19 pandemic has severely affected the public bus occupancy rates, which would have significantly increased the emissions per PKT of public buses in Taichung City.

**Fig. 2:**
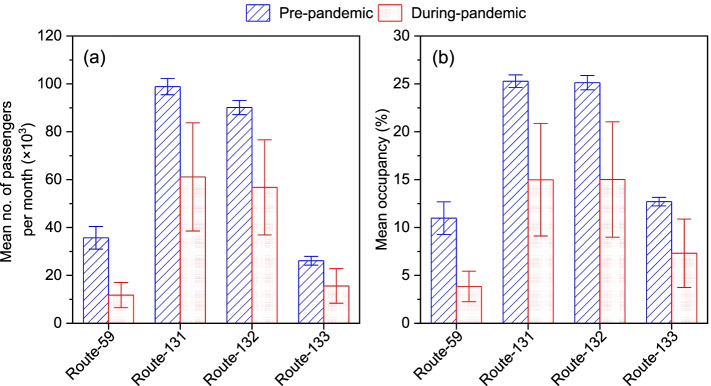
Mean ± SD of the monthly ridership (**a**) and the monthly bus occupancy rate (**b**)

The monthly total ridership (number of passengers taking the bus ride) and the monthly total actual PKT for the entire study period for each bus route are presented in Table S6. Likewise, the pre-pandemic and during-pandemic mean monthly ridership and occupancy rates are presented in Fig. 2. As Fig. 2a depicts, the pre-pandemic mean monthly ridership was 35,686 ± 4723, while the during-pandemic mean monthly ridership was 11,786 ± 5253 on the route-59. The during-pandemic mean monthly ridership was just about one-third (33%) of the pre-pandemic mean monthly ridership. Likewise, on route-131, the during-pandemic mean monthly ridership (61,145 ± 22,593) was 62% of that before the pandemic (98,870 ± 3411). On route-132, the during-pandemic monthly ridership (56,780 ± 19,883) was 63% of that before the pandemic (90,131 ± 2980). Similarly, on route-133, the during-pandemic monthly ridership (15,590 ± 7227) was 60% of that before the pandemic (26,130 ± 1823). These data show that the mean monthly ridership decreased by a minimum of 37% (route-132) to the highest of 67% (route-59). Comparable to our results, a prior study related to the 2003-SARS epidemic reported that the daily subway ridership dropped by approximately 50% during the peak of the epidemic in Taiwan [14]. Likewise, in Hanoi, Vietnam, the first wave of the COVID-19 pandemic caused the public bus ridership to drop by approximately 30% in February 2020 and by 96% in April 2021 compared to that in January 2020 [12]. In Qingdao, China, the public bus ridership has been estimated to drop by approximately 56% during the COVID-19 pandemic periods [2], which was quite close to our results (37–67% drop in bus ridership).

Likewise, as shown in Fig. 2b, the mean monthly occupancy rates before the pandemic were 10.9 ± 1.7%, 25.3 ± 0.7%, 25.1 ± 0.7%, and 12.7 ± 0.4% on the routes-59, 131, 132, and 133, respectively. The during-pandemic mean monthly ridership on the routes-59, 131, 132, and 133 were 3.8 ± 1.6%, 15.0 ± 5.9%, 15.0 ± 6.0%, and 7.3 ± 3.6%, respectively. These results showed that the mean occupancy rates during the pandemic decreased by 40 to 65% from the pre-pandemic occupancy rates. Independent sample *t*-tests showed that the mean monthly occupancy rates during the pandemic were significantly different from the mean monthly occupancy rates before the pandemic on all four test routes at a 0.01 level of significance (Table S7). Therefore, it can be concluded that the public bus occupancy rates were significantly affected by the COVID-19 pandemic in Taichung City.

The average distance traveled by a passenger per bus trip was also calculated for each month and bus routes (Table S8). Before the pandemic, the average distances traveled by a passenger on a bus ride were 4.11 ± 0.03, 6.13 ± 0.52, 5.13 ± 0.03, and 4.44 ± 0.09 km, on the bus routes-59, 131, 132, and 133, respectively. Likewise, the average distances traveled by a passenger on a bus ride during the pandemic were 4.40 ± 0.14, 5.91 ± 0.77, 4.97 ± 0.93, and 4.29 ± 0.77 km on the bus routes-59, 131, 132, and 133, respectively. The average distance traveled per bus ride was only marginally lower during the pandemic than before the pandemic, except on route-59. These results suggested that despite the significant drop in the public bus ridership and occupancy rates during the pandemic, the average distance traveled by a passenger in a bus ride would not change significantly.

### Real-world emission factors of gaseous pollutants

The real-world emission factors of gaseous pollutants and FC rates of the bus and car measured in the present study are summarized in Table 2. It should be noted that the bus emissions were measured for the route-59 bus only. As Table 2 depicts, the bus emission factors, especially CO, THC, and CO_2_, measured in the present study were relatively less than those reported by prior studies [4, 31]. The difference in emission factors might be attributed to the difference in driving dynamics, road attributes, engine characteristics, passenger capacity, and passenger loads. For example, in the study by Rosero et al. [31], the reported emission factors represent 0–1200 kg passenger loads, while in the present study, the emissions were measured without passenger loads. Likewise, the passenger capacity of the bus in the present study (65) was also less than that in the study conducted by Rosero et al. [31]. In contrast, the mean NO emission factor for the bus obtained in the present study was comparable to that reported in prior studies [4, 31]. Likewise, Wang et al. [32] reported the real-world emission factors of CO, THC, NO_*x*_, and CO_2_, as well as FC rates for four diesel buses belonging to either Euro III or IV emission certification levels and of engine displacement volumes ranging from 5900 to 6700 cm^3^, and the emission factors were comparable to those in the present study. Likewise, the mean distance-specific emission factors for the car measured in the present study have been compared with those reported in the literature (Table 2). The real-world CO emission factor of the car (0.58 ± 0.18 g km^− 1^) was slightly higher than those reported by Yang et al. [33] for the car of comparable engine size. However, the emission factors of THC, NO, CO_2_, and FC rates for the car in the present study were similar to those reported by Yang et al. [33]. In contrast, the emission factors and FC rates of the car obtained in the present study were much less than those reported for Taiwan Phase-1 to 3 passenger cars [34]. This difference might be largely due to the difference in emission certification levels of cars in the two studies as a relatively new car was used in the present study.

**Table 2:** Real-world distance-specific emission factors and FC of the test vehicles

Parameters	CO (g km^**− 1**^)	THC (mg km^**− 1**^)	NO (g km^**− 1**^)	CO_**2**_ (g km^**− 1**^)	FC (g km^**− 1**^)
Bus (this study)	1.61 ± 1.02	41 ± 21	13 ± 1.5	591 ± 47.3	188 ± 15
Bus [31] ^a^	4.52 ± 1.33	226 ± 16	12 ± 1.3 ^b^	1970 ± 183	534 ± 48 ^c^
Bus [4] ^d^	6.78	NA ^e^	13 ^b^	NA ^e^	NA ^e^
Bus [32] ^f^	1.31–6.70	38–191	10–13 ^b^	799–1128	256–358
Car (this study)	0.58 ± 0.18	57 ± 13	0.058 ± 0.006	208 ± 20	66 ± 6.4
Car [33] ^g^	0.26–2.34	68–101	0.020–0.084 ^b^	NA ^e^	57–84 ^h^
Car [34] ^i^	1.52–2.14	150–210	0.06–0.38	231–248	61–83 ^h^

^a^ Euro V diesel bus with a 7790 cm^3^ engine and the passenger capacity of 78

^b^ Emission factor for NO_*x*_

^c^ Converted from L km^− 1^ assuming density of diesel as 851 g L^− 1^

^d^ Diesel bus (King Long) 10.2 t capacity; modeled emission factors

^e^ Not available

^f^ Euro III and IV diesel buses with 5900–6700 cm^3^ engine and equipped with either SCR or no tailpipe emission control devices; real-world emission measurement

^g^ Gasoline cars with 1794–2354 cm^3^ engine; emissions tested on a chassis dynamometer

^h^ Converted from L km^− 1^ assuming density of gasoline as 757 g L^− 1^

^i^ Gasoline cars with 1300–2000 cm^3^ engine equipped with TWC and belonging to Taiwan Phase-1 to 3; emissions tested on a chassis dynamometer

### Effects of COVID-19 pandemic on emissions per PKT

Table 3 presents the comparison of air pollutant emissions and FC per PKT of the bus before and during the COVID-19 pandemic. Since the bus emissions were measured for the route-59 bus, the emissions per PKT were also calculated for the route-59 buses only using the passenger occupancy rates on the buses of the same route. Results showed that the mean during-pandemic emissions and FC per PKT were notably higher than both the pre-pandemic emissions per PKT, as well as reference emissions per PKT for the bus. The during-pandemic mean emissions per PKT were more than 3.5 times the pre-pandemic mean emissions per PKT and more than 32 times the reference emissions per PKT. A study conducted in Qingdao, China, estimated that the during-pandemic CO_2_ emission per PKT of public buses was > 2 times the pre-pandemic CO_2_ emission per PKT [2]. Their result is similar to that obtained in the present study.

**Table 3:** Emissions of gaseous pollutants and FC per PKT of bus, car, and motorcycle

Parameter	Unit	Bus			Car	Motorcycle ^**b**^
		Pre-pandemic	During-pandemic	Reference ^**a**^		
CO	g PKT^−1^	0.23 ± 0.03	0.81 ± 0.56	0.02	0.29	3.10
THC	mg PKT^−1^	5.9 ± 0.8	21 ± 14	0.64	29	876
NO	g PKT^−1^	1.9 ± 0.3	6.6 ± 4.6	0.20	0.03	0.23
CO_2_	g PKT^−1^	84 ± 11	298 ± 206	9	104	60
FC	g PKT^−1^	27 ± 4	95 ± 66	3	33	22

^a^ Emissions per PKT when the bus occupancy is 100%

^b^ Fleet-average emission factors compiled from the literature

Moreover, the pre-pandemic emissions per PKT were also more than 9 times the reference emissions per PKT, which was because the public buses in Taichung City were largely unoccupied on most of the trips, even before the pandemic. A prior study also reported that the emissions per PKT of public buses were significantly affected by the change in passenger ridership at different traffic hours [4]. For example, CO and NO_*x*_ emissions per PKT of public buses during lean traffic hours were as high as two times those during peak traffic hours [4].

The mean pre-pandemic CO emission of the bus (0.23 ± 0.03 g PKT^− 1^) was less than the CO emission of the car (0.29 g PKT^− 1^). But the during-pandemic CO emission of the bus (0.81 ± 0.56 g PKT^− 1^) was much higher than the CO emission of the car. Therefore, based on the CO emission per PKT, the bus was less polluting than the car before the COVID-19 pandemic, which became more polluting than the car during the pandemic. However, irrespective of the pandemic scenario, the bus was much cleaner than the motorcycle based on the CO emissions per PKT. Likewise, the mean THC emissions of the bus were 5.9 ± 0.8 mg PKT^− 1^ (pre-pandemic) and 21 ± 14 mg PKT^− 1^ (during-pandemic). These values were notably less than the THC emissions per PKT of the car and motorcycles. Therefore, in terms of THC emissions, public buses can be much cleaner, even with very low occupancy rates during the pandemic, than cars and motorcycles. This was because the diesel engine of buses typically emits less THC than gasoline engines of cars and motorcycles.

Unlike CO and THC, the mean NO emission per PKT of the bus was much higher than those of cars and motorcycles, irrespective of the pandemic scenario. Diesel engines typically operate at high temperatures than gasoline engines, which usually causes the diesel buses to emit a higher amount of NO than gasoline cars and motorcycles. Similar to the results obtained in the present study, a prior study also reported that public buses had a higher NO_*x*_ emission per PKT than passenger cars [4]. Likewise, the mean CO_2_ emission of the bus before the pandemic (84 ± 11 g PKT^− 1^) was less than that of the car (104 g PKT^− 1^), indicating that the public buses can be desirable over private cars based on CO_2_ emissions at the normal bus occupancy rates typical of Taichung City. However, during the pandemic, the CO_2_ emission from the bus (298 ± 206 g PKT^− 1^) became significantly higher than that of cars (104 g PKT^− 1^) and motorcycles (60 g PKT^− 1^). The results for FC were similar to those for CO_2_. A prior study conducted in a Chinese city also suggested that the bus emission of CO_2_ per PKT can be higher than those of cars during the lean traffic hours in the COVID-19 pandemic period [2].

To summarize, based on CO and THC emissions per PKT, public buses might be less polluting than gasoline cars and motorcycles, even at low passenger occupancy rates in the public buses. However, the emission benefit from public buses in terms of NO and CO_2_ may be uncertain when the bus occupancy rates are low, as observed in Taichung City buses. Although many recent studies showed improved ambient air quality, a positive environmental implication, due to restricted mobility during the pandemic [9, 15–17, 19], our analysis of tailpipe emissions per PKT of buses suggested that the benefit of public buses in terms of NO and CO_2_ emissions per PKT over private modes of transportation became doubtful in the context of the COVID-19 pandemic. In other words, public bus transit systems can be less efficient in terms of emissions per PKT than private transport modes in the changing scenario due to the COVID-19 pandemic if appropriate measures are not adopted to increase bus usage.

### Estimated break-even occupancy rates for buses

First of all, the minimum emission per PKT of private modes of transportation (car and motorcycle) was taken from the data presented in Table 3. For example, the minimum CO emission per PKT for private transport modes was 0.29 g PKT^− 1^, which was observed for the car (Table 3). Likewise, the minimum emissions for private modes of transport were 29 mg PKT^− 1^ for THC (car), 0.03 g PKT^− 1^ for NO (car), 60 g PKT^− 1^ for CO_2_ (motorcycle), and 22 g PKT^− 1^ for FC (motorcycle). Now, if the bus is less emitting than cars and motorcycles, then the minimum emissions per PKT mentioned above should be the maximum emission threshold for the bus. This emission threshold would be achieved by the bus at the corresponding threshold occupancy rates or break-even occupancy rates. The break-even occupancy rates for the bus are presented in Table 4. The results showed that the bus would be emitting less amount of CO per PKT than the cars and motorcycles when the bus occupancy rate is > 9.2%. Likewise, at occupancy rates above 3.1%, the bus would emit less amount of THC per PKT than cars and motorcycles. Similarly, the break-even occupancy rates were obtained as 15.4% for CO_2_ and 13.8% for FC. However, the difference in the emission factors of NO between the bus and car was so huge that the bus would emit more NO per PKT at or even above the 100% occupancy rate. Therefore, it might not be possible to achieve less NO emission per PKT for diesel buses than for gasoline cars and motorcycles when the engine and emission control technologies of the buses, cars, and motorcycles are similar to those in the present study. To summarize, at the occupancy rates > 15.4%, public buses would be emitting less amount of CO, THC, and CO_2_, but not NO, and consuming less amount of fuel per PKT than the private modes of transportation (cars and motorcycles). The NO emission factor of the bus at the occupancy rate of 15.4% would be 1.3 g PKT^− 1^, which is higher than the NO emissions factor per PKT of motorcycles and cars. However, as shown in Fig. 1, the occupancy rates of the buses on route-59 were always less than 15%. Therefore, the public transport management authorities may be required to develop and implement strategies to increase the passenger occupancy rates in public buses in Taichung City.

**Table 4:** The minimum threshold bus occupancy rates

Parameters	CO	THC	NO	CO_**2**_	FC
Threshold emission per PKT (minimum from private modes) ^a^	0.29	29	0.03	60	22
Mean distance-specific emission factor of bus ^b^	1.6	41	13	591	188
Threshold passenger number for the bus ^c^	6	2	434	10	9
Maximum passenger capacity of the bus	65	65	65	65	65
Threshold occupancy rate (%)	9.2	3.1	668	15.4	13.8

^a^ Unit for THC is mg PKT^−1^ and that for CO, NO, CO_2_ and FC is g PKT^− 1^

^b^ Unit for THC is mg km^− 1^ and that for CO, NO, CO_2_ and FC is g km^− 1^

^c^ Rounded-up values

It should be noted that the bus and car emission factors obtained in the present study might not represent the mixed fleet of buses and cars having different engine and emission control technologies. Therefore, the results obtained in the present case study should be taken as indicative of the probable scenario. Similar future studies might be conducted with a better representation of the bus, car, and motorcycle fleets.

## Conclusions

This study compared the monthly public bus occupancy rates in Taichung City, Taiwan, before and during the COVID-19 pandemic. It also investigated the effect of reduced passenger occupancy on real-world emissions of regulated gaseous pollutants per PKT. Our results showed that the mean public bus occupancy rates were 11–25% before the pandemic and 4–15% during the pandemic on different bus routes. The mean monthly occupancy rates during the pandemic were significantly different (*p* < 0.01) from the mean monthly occupancy rates before the pandemic on all test routes, indicating that the pandemic has significantly affected the public bus occupancy in Taichung City. Moreover, buses were occupied only up to 25% of the total passenger capacity, even before the pandemic, which suggested that the bus ridership should be increased to improve the environmental performance and efficiency of the public bus transit system. The analysis of emissions per PKT indicated that the public bus was less polluting than the private car and motorcycles, based on CO and THC emissions. However, NO and CO_2_ emissions per PKT of the public bus were much higher than the respective emissions per PKT of the car and motorcycle during the COVID-19 pandemic, consequently making the environmental benefits of public buses over the private modes of transportation uncertain. Moreover, the analysis of break-even bus occupancy rate showed that public buses could achieve less emission per PKT of CO, THC, and CO_2_ than gasoline cars and motorcycles when the bus occupancy rates are above 15%. Below the break-even occupancy rates, the emission benefits of public buses over private cars and motorcycles would be overturned. Therefore, our findings suggested a need to optimize the public bus route designs, bus frequencies, and implementation of anti-pandemic measures in the changing scenario caused by the COVID-19 pandemic so as to increase the public bus ridership and maximize the environmental benefits of the public bus transit systems over private modes of transportation.

## Supplementary Information

**Additional file 1: Table S1.** Timeline of COVID-19 pandemic alert levels in Taiwan. **Table S2.** Total number of bus trips on the sample bus routes in different months. **Table S3.** Moisture correction factors. **Table S4.** Emission factors of motorcycles belonging to various emission certification levels of Taiwan. **Table S5.** Fleet-average emission factor for Taiwan’s 2021 motorcycle fleet. **Table S6.** Monthly total ridership and PKT on the sample bus routes in different months. **Table S7.** Independent sample t-test of monthly average bus occupancy rates between pre-pandemic and during-pandemic scenarios. **Table S8.** Average distance (km) traveled by a passenger on a bus ride. **Fig. S1.** Map of the study area showing the sample bus routes. **Fig. S2.** Schematic of the study design.

## Data Availability

All data generated or analyzed during this study are provided in the submitted article.

## References

[1] Guzman LA, Oviedo D (2018). Accessibility, affordability and equity: assessing 'pro-poor' public transport subsidies in Bogota. Transport Policy..

[2] Sui Y, Zhang HR, Shang WL, Sun RC, Wang CY, Ji J, et al. Mining urban sustainable performance: spatio-temporal emission potential changes of urban transit buses in post-COVID-19 future. Appl Energ. 2020;280:115966. 10.1016/j.apenergy.2020.115966 PMC7544736 33052166

[3] Bigazzi A. Marginal emission factors for public transit: effects of urban scale and density. Transport Res D-Tr E. 2020;88:102585. 10.1016/j.trd.2020.102585 PMC7580529 33110387

[4] Chen XH, Shan XN, Ye JH, Yi FB, Wang YF (2017). Evaluating the effects of traffic congestion and passenger load on feeder bus fuel and emissions compared with passenger car. Transp Res Proc..

[5] Marra AD, Sun LH, Corman F (2022). The impact of COVID-19 pandemic on public transport usage and route choice: evidences from a long-term tracking study in urban area. Transport Policy..

[6] Sogbe E (2021). The evolving impact of coronavirus (COVID-19) pandemic on public transportation in Ghana. Case Stud Transp Pol..

[7] Czodorova R, Dockalik M, Gnap J (2021). Impact of COVID-19 on bus and urban public transport in SR. Transp Res Proc..

[8] Advani M, Sharma N, Dhyani R (2021). Mobility change in Delhi due to COVID and its' immediate and long term impact on demand with intervened non motorized transport friendly infrastructural policies. Transport Policy..

[9] Sahraei MA, Kuskapan E, Codur MY (2021). Public transit usage and air quality index during the COVID-19 lockdown. J Environ Manage..

[10] Hadei M, Mohebbi SR, Hopke PK, Shahsavani A, Bazzazpour S, Alipour M (2021). Presence of SARS-CoV-2 in the air of public places and transportation. Atmos Pollut Res..

[11] Nguyen MH, Pojani D (2021). Covid-19 need not spell the death of public transport: learning from Hanoi’s safety measures. J Transp Health..

[12] Nguyen MH, Pojani D. Why are Hanoi students giving up on bus ridership? Transportation. 2022. 10.1007/s11116-021-10262-9 PMC8762445 35068615

[13] MOTC (2022). Transportation Statistics.

[14] Wang KY (2014). How change of public transportation usage reveals fear of the SARS virus in a city. PLoS One..

[15] Albayati N, Waisi B, Al-Furaiji M, Kadhom M, Alalwan H (2021). Effect of COVID-19 on air quality and pollution in different countries. J Transp Health..

[16] Cooper MJ, Martin RV, Hammer MS, Levelt PF, Veefkind P, Lamsal LN (2022). Global fine-scale changes in ambient NO_2_ during COVID-19 lockdowns. Nature..

[17] He GJ, Pan YH, Tanaka T (2020). The short-term impacts of COVID-19 lockdown on urban air pollution in China. Nat Sustain..

[18] Briz-Redon A, Belenguer-Sapina C, Serrano-Aroca A (2021). Changes in air pollution during COVID-19 lockdown in Spain: a multi-city study. J Environ Sci-China..

[19] Wen LY, Yang C, Liao XL, Zhang YH, Chai XY, Gao WJ (2022). Investigation of PM_2.5_ pollution during COVID-19 pandemic in Guangzhou, China. J Environ Sci-China..

[20] Ritchie H, Mathieu E, Rodes-Guirao L, Appel C, Giattino C, Ortiz-Ospina E, et al. Taiwan: Coronavirus Pandemic Country Profile. OurWorldInData.org; 2020. Accessed 29 Jan 2022.

[21] Schafer AW, Yeh SN (2020). A holistic analysis of passenger travel energy and greenhouse gas intensities. Nat Sustain..

[22] Yang HH, Dhital NB, Cheruiyot NK, Wang LC, Wang SX (2021). Effects of road grade on real-world tailpipe emissions of regulated gaseous pollutants and volatile organic compounds for a Euro 5 motorcycle. Atmos Pollut Res..

[23] Giechaskiel B, Zardini AA, Clairotte M (2019). Exhaust gas condensation during engine cold start and application of the dry-wet correction factor. Appl Sci-Basel..

[24] Yu Q, Li TZ, Li H (2016). Improving urban bus emission and fuel consumption modeling by incorporating passenger load factor for real world driving. Appl Energy..

[25] Tsai JH, Yao YC, Huang PH, Chiang HL (2017). Criteria pollutants and volatile organic compounds emitted from motorcycle exhaust under various regulation phases. Aerosol Air Qual Res..

[26] Tsai JH, Yao YC, Huang PH, Chiang HL (2018). Fuel economy and volatile organic compound exhaust emission for motorcycles with various running mileages. Aerosol Air Qual Res..

[27] Tsai JH, Huang PH, Chiang HL (2014). Characteristics of volatile organic compounds from motorcycle exhaust emission during real-world driving. Atmos Environ..

[28] Yao YC, Tsai JH, Wang IT (2013). Emissions of gaseous pollutant from motorcycle powered by ethanol-gasoline blend. Appl Energy..

[29] MOTC (2021). Motor Vehicle Registrations.

[30] Wei TC, Frey HC (2022). Intermodal comparison of tailpipe emission rates between transit buses and private vehicles for on-road passenger transport. Atmos Environ..

[31] Rosero F, Fonseca N, Lopez JM, Casanova J (2021). Effects of passenger load, road grade, and congestion level on real-world fuel consumption and emissions from compressed natural gas and diesel urban buses. Appl Energy..

[32] Wang AJ, Ge YS, Tan JW, Fu ML, Shah AN, Ding Y (2011). On-road pollutant emission and fuel consumption characteristics of buses in Beijing. J Environ Sci-China..

[33] Yang HH, Dhital NB, Wang LC, Hsieh YS, Lee KT, Hsu YT (2019). Chemical characterization of fine particulate matter in gasoline and diesel vehicle exhaust. Aerosol Air Qual Res..

[34] Chiang HL, Tsai JH, Yao YC, Ho WY (2008). Deterioration of gasoline vehicle emissions and effectiveness of tune-up for high-polluted vehicles. Transport Res D-Tr E..

