# Supplementary material for: Effects of the COVID-19 pandemic on public bus occupancy and real-world tailpipe emissions of gaseous pollutants per passenger kilometer traveled
Source: Sustain Environ Res. 2022 Aug 9;32(1):35. doi: 10.1186/s42834-022-00146-7 (PMC9361239; doi:10.1186/s42834-022-00146-7)
Supplement: Supplementary file 1 — Additional file 1: Table S1. Timeline of COVID-19 pandemic alert levels in Taiwan. Table S2. Total number of bus trips on the sample bus routes in different months. Table S3. Moisture correction factors. Table S4. Emission factors of motorcycles belonging to various emission certification levels of Taiwan. Table S5. Fleet-average emission factor for Taiwan’s 2021 motorcycle fleet. Table S6. Monthly total ridership and PKT on the sample bus routes in different months. Table S7. Independent sample t-test of monthly average bus occupancy rates between pre-pandemic and during-pandemic scenarios. Table S8. Average distance (km) traveled by a passenger on a bus ride. Fig. S1. Map of the study area showing the sample bus routes. Fig. S2. Schematic of the study design. [file 42834_2022_146_MOESM1_ESM.docx]

**Supplementary materials**

**Table S1** Timeline of COVID-19 pandemic alert levels in Taiwan

| **Timeline** | **Alert levels** |
| --- | --- |
| Until 2020-01-20 | No restrictions (Pre-pandemic) |
| 2020-01-21 to 2021-05-10 | Level-1 ^a^ |
| 2021-05-11 to 2021-05-18 | Level-2 ^b^ |
| 2021-05-19 to 2021-07-26 | Level-3 ^c^ |
| 2021-07-27 to 2021-12-13 | Level-2 |

^a^ Sporadic community infections caused by overseas immigration (basic control measures, such as social distancing).

^b^ Local cases of infection with unknown origins (moderate level of restrictions, such as banning outdoor gatherings of 500 or more people, or indoor gatherings of 100 or more people).

^c^ More than ten local cases of infection with unknown origins in a day (stringent restrictions, such as banning outdoor gatherings of ten or more people, or indoor gatherings of five or more people).

**Table S2** Total number of bus trips on the sample bus routes in different months

| **Months** | **Route-59** | **Route-131** | **Route-132** | **Route-133** |
| --- | --- | --- | --- | --- |
| Sep-2019 | 880 | 1740 | 1750 | 1000 |
| Oct-2019 | 920 | 1814 | 1903 | 1060 |
| Nov-2019 | 888 | 1752 | 1839 | 1020 |
| Dec-2019 | 920 | 1814 | 1903 | 1060 |
| Jan-2020 | 920 | 1814 | 1815 | 1060 |
| Feb-2020 | 848 | 1678 | 1690 | 960 |
| Mar-2020 | 920 | 1814 | 1903 | 1060 |
| Apr-2020 | 880 | 1740 | 1830 | 1000 |
| May-2020 | 912 | 1802 | 1894 | 1040 |
| Jun-2020 | 888 | 1752 | 1755 | 1020 |
| Jul-2020 | 928 | 1826 | 1820 | 1080 |
| Aug-2020 | 912 | 1802 | 1810 | 1040 |
| Sep-2020 | 904 | 1776 | 1765 | 1060 |
| Oct-2020 | 896 | 1778 | 1876 | 1000 |
| Nov-2020 | 888 | 1752 | 1839 | 1020 |
| Dec-2020 | 928 | 1826 | 1912 | 1080 |
| Jan-2021 | 904 | 1790 | 1805 | 1020 |
| Feb-2021 | 800 | 1592 | 1620 | 880 |
| Mar-2021 | 920 | 1814 | 1903 | 1060 |
| Apr-2021 | 872 | 1728 | 1821 | 860 |
| May-2021 | 912 | 1802 | 1894 | 1040 |
| Jun-2021 | 888 | 1752 | 1755 | 1020 |
| Jul-2021 | 920 | 1814 | 1815 | 1060 |
| Aug-2021 | 920 | 1814 | 1815 | 1060 |
| Sep-2021 | 888 | 1752 | 1755 | 1020 |
| Mean ± SD | 898 ± 29 | 1774 ± 53 | 1819 ± 73 | 1023 ± 54 |

**Table S3** Moisture correction factors

| **Sample** | **Moisture correction factor (mean ± SD)** |
| --- | --- |
| Bus-1 | 0.96 ± 0.02 |
| Bus-2 | 0.95 ± 0.02 |
| Bus-3 | 0.95 ± 0.02 |
| Bus-4 | 0.95 ± 0.02 |
| Car-1 | 0.86 ± 0.002 |
| Car-2 | 0.86 ± 0.002 |
| Car-3 | 0.86 ± 0.002 |
| Car-4 | 0.86 ± 0.001 |

**Table S4** Emission factors of motorcycles belonging to various emission certification levels of Taiwan

| **Emission phase** | **Model year** | **CO**  **(g km^-1^)** | **THC**  **(mg km^-1^)** | **NO**  **(g km^-1^)** | **CO_2_**  **(g km^-1^)** | **FC**  **(g km^-1^)** | **Source** |
| --- | --- | --- | --- | --- | --- | --- | --- |
| Phase-3 | 2003 or older | 5.81 | 1840 | 0.20 | 61 | 25 | [1] ^a^ |
|  |  | 7.32 | 1831 | 0.19 | 62 | 26 | [2] ^a^ |
| Phase-4 | 2004-2006 | 3.85 | 900 | 0.21 | 61 | 23 | [1] ^a^ |
|  |  | 3.83 | 982 | 0.20 | 61 | 23 | [2] ^a^ |
| Phase-5 | 2007-2016 | 2.45 | 840 | 0.23 | 60 | 22 | [1] ^a^ |
|  |  | 2.42 | 836 | 0.23 | 61 | 22 | [2] ^a^ |
|  |  | 2.84 | 585 | 0.30 | − | − | [3] ^b^ |
|  |  | 4.76 | 3397 | 0.09 | 58 | 22 | [4] ^c^ |
| Phase-7 | 2021 or newer | 0.30 | 9 | 0.01 | 41 | 13 | [5] ^d^ |

^a^ Dynamometer test with the ECE test cycle.

^b^ Average of fuel injection and carburetor motorcycles; dynamometer test.

^c^ Average of emissions on three test routes, real-world emission test.

^d^ Average of emissions on two routes, real-world emission test.

**Table S5** Fleet-average emission factor for Taiwan’s 2021 motorcycle fleet

| **Model year** | **Taiwan’s 2021 motorcycle fleet composition (%) [6]** | **CO**  **(g km^-1^)** | **THC**  **(mg km^-1^)** | **NO**  **(g km^-1^)** | **CO_2_**  **(g km^-1^)** | **FC**  **(g km^-1^)** |
| --- | --- | --- | --- | --- | --- | --- |
| 2003 and older | 13.5 | 6.57 | 1835 | 0.20 | 62 | 25 |
| 2004-2006 | 9.1 | 3.84 | 941 | 0.21 | 61 | 23 |
| 2007-2020 | 71.9 | 2.57 | 754 | 0.25 | 60 | 22 |
| 2021 and newer | 5.5 | 0.30 | 9 | 0.01 | 41 | 13 |
| Motorcycle fleet | 100 | 3.10 | 876 | 0.23 | 60 | 22 |

**Table S6** Monthly total ridership and PKT on the sample bus routes in different months

| **Months** | **Route-59** |  | **Route-131** |  | **Route-132** |  | **Route-133** |  |
| --- | --- | --- | --- | --- | --- | --- | --- | --- |
|  | **Ridership** | **PKT** | **Ridership** | **PKT** | **Ridership** | **PKT** | **Ridership** | **PKT** |
| Sep-19 | 42688 | 176772 | 95936 | 518837 | 89345 | 453639 | 23676 | 106159 |
| Oct-19 | 33247 | 136924 | 100940 | 616149 | 93818 | 481388 | 27945 | 120419 |
| Nov-19 | 32526 | 132087 | 96012 | 613579 | 86654 | 446790 | 25976 | 116224 |
| Dec-19 | 34283 | 141373 | 102592 | 678836 | 90708 | 466704 | 26922 | 121424 |
| Jan-20 | 29317 | 122290 | 75156 | 506589 | 67302 | 329940 | 17348 | 82791 |
| Feb-20 | 14997 | 63932 | 53973 | 351217 | 46476 | 216593 | 9898 | 45155 |
| Mar-20 | 13397 | 57363 | 78233 | 239884 | 72036 | 91728 | 23080 | 23600 |
| Apr-20 | 11569 | 48952 | 65457 | 434404 | 58612 | 295744 | 19180 | 84658 |
| May20 | 12242 | 51340 | 75808 | 504682 | 67840 | 344590 | 21538 | 93072 |
| Jun-20 | 12842 | 55426 | 74820 | 495049 | 66793 | 333803 | 20074 | 86806 |
| Jul-20 | 12768 | 55451 | 68646 | 443327 | 57876 | 273349 | 13702 | 62150 |
| Aug-20 | 13391 | 58712 | 70240 | 456106 | 55792 | 264479 | 13614 | 62424 |
| Sep-20 | 13006 | 56203 | 71836 | 422046 | 69826 | 396952 | 20513 | 92142 |
| Oct-20 | 13230 | 59314 | 81115 | 478800 | 73065 | 424023 | 21590 | 96993 |
| Nov-20 | 13371 | 59300 | 82709 | 476620 | 76412 | 437606 | 22662 | 102858 |
| Dec-20 | 13312 | 58033 | 92523 | 542389 | 84027 | 463780 | 25098 | 110504 |
| Jan-21 | 11494 | 51108 | 67279 | 395594 | 64137 | 354090 | 18331 | 82691 |
| Feb-21 | 9344 | 40519 | 46933 | 282037 | 47154 | 254886 | 11438 | 53207 |
| Mar-21 | 12765 | 57336 | 74225 | 429828 | 75562 | 418969 | 23647 | 103806 |
| Apr-21 | 11998 | 52801 | 69288 | 394972 | 70329 | 376185 | 19295 | 92780 |
| May21 | 6966 | 31259 | 41479 | 231008 | 40874 | 218868 | 10451 | 44569 |
| Jun-21 | 2560 | 11789 | 10164 | 55971 | 10625 | 49487 | 1938 | 7544 |
| Jul-21 | 3760 | 17354 | 15345 | 84419 | 16701 | 78960 | 3427 | 14145 |
| Aug-21 | 6326 | 29524 | 25551 | 144943 | 28965 | 138663 | 5287 | 23478 |
| Sep-21 | 8843 | 39895 | 43262 | 257374 | 41979 | 203258 | 5287 | 23478 |

**Table S7** Independent sample t-test of monthly average bus occupancy rates between pre-pandemic and during-pandemic scenarios

| **Route** | ***p*-value** | **Remarks** |
| --- | --- | --- |
| 59 | 0.000 | Mean bus occupancy rates between pre-pandemic and during-pandemic scenarios are significantly different at 0.01 level |
| 131 | 0.002 | Mean bus occupancy rates between pre-pandemic and during-pandemic scenarios are significantly different at 0.01 level |
| 132 | 0.003 | Mean bus occupancy rates between pre-pandemic and during-pandemic scenarios are significantly different at 0.01 level |
| 133 | 0.007 | Mean bus occupancy rates between pre-pandemic and during-pandemic scenarios are significantly different at 0.01 level |

**Table S8** Average distance (km) traveled by a passenger on a bus ride

| **Months** | **Route-59** | **Route-131** | **Route-132** | **Route-133** |
| --- | --- | --- | --- | --- |
| 19-Sep | 4.14 | 5.41 | 5.08 | 4.48 |
| 19-Oct | 4.12 | 6.10 | 5.13 | 4.31 |
| 19-Nov | 4.06 | 6.39 | 5.16 | 4.47 |
| 19-Dec | 4.12 | 6.62 | 5.15 | 4.51 |
| 20-Jan | 4.17 | 6.74 | 4.90 | 4.77 |
| 20-Feb | 4.26 | 6.51 | 4.66 | 4.56 |
| 20-Mar | 4.28 | 3.07 | 1.27 | 1.02 |
| 20-Apr | 4.23 | 6.64 | 5.05 | 4.41 |
| 20-May | 4.19 | 6.66 | 5.08 | 4.32 |
| 20-Jun | 4.32 | 6.62 | 5.00 | 4.32 |
| 20-Jul | 4.34 | 6.46 | 4.72 | 4.54 |
| 20-Aug | 4.38 | 6.49 | 4.74 | 4.59 |
| 20-Sep | 4.32 | 5.88 | 5.68 | 4.49 |
| 20-Oct | 4.48 | 5.90 | 5.80 | 4.49 |
| 20-Nov | 4.43 | 5.76 | 5.73 | 4.54 |
| 20-Dec | 4.36 | 5.86 | 5.52 | 4.40 |
| 21-Jan | 4.45 | 5.88 | 5.52 | 4.51 |
| 21-Feb | 4.34 | 6.01 | 5.41 | 4.65 |
| 21-Mar | 4.49 | 5.79 | 5.54 | 4.39 |
| 21-Apr | 4.40 | 5.70 | 5.35 | 4.81 |
| 21-May | 4.49 | 5.57 | 5.35 | 4.26 |
| 21-Jun | 4.60 | 5.51 | 4.66 | 3.89 |
| 21-Jul | 4.62 | 5.50 | 4.73 | 4.13 |
| 21-Aug | 4.67 | 5.67 | 4.79 | 4.44 |
| 21-Sep | 4.51 | 5.95 | 4.84 | 4.44 |
| Pre-pandemic mean ± SD | 4.11 ± 0.03 | 6.13 ± 0.52 | 5.13 ± 0.03 | 4.44 ± 0.09 |
| During-pandemic mean ± SD | 4.40 ± 0.14 | 5.91 ± 0.77 | 4.97 ± 0.93 | 4.29 ± 0.77 |


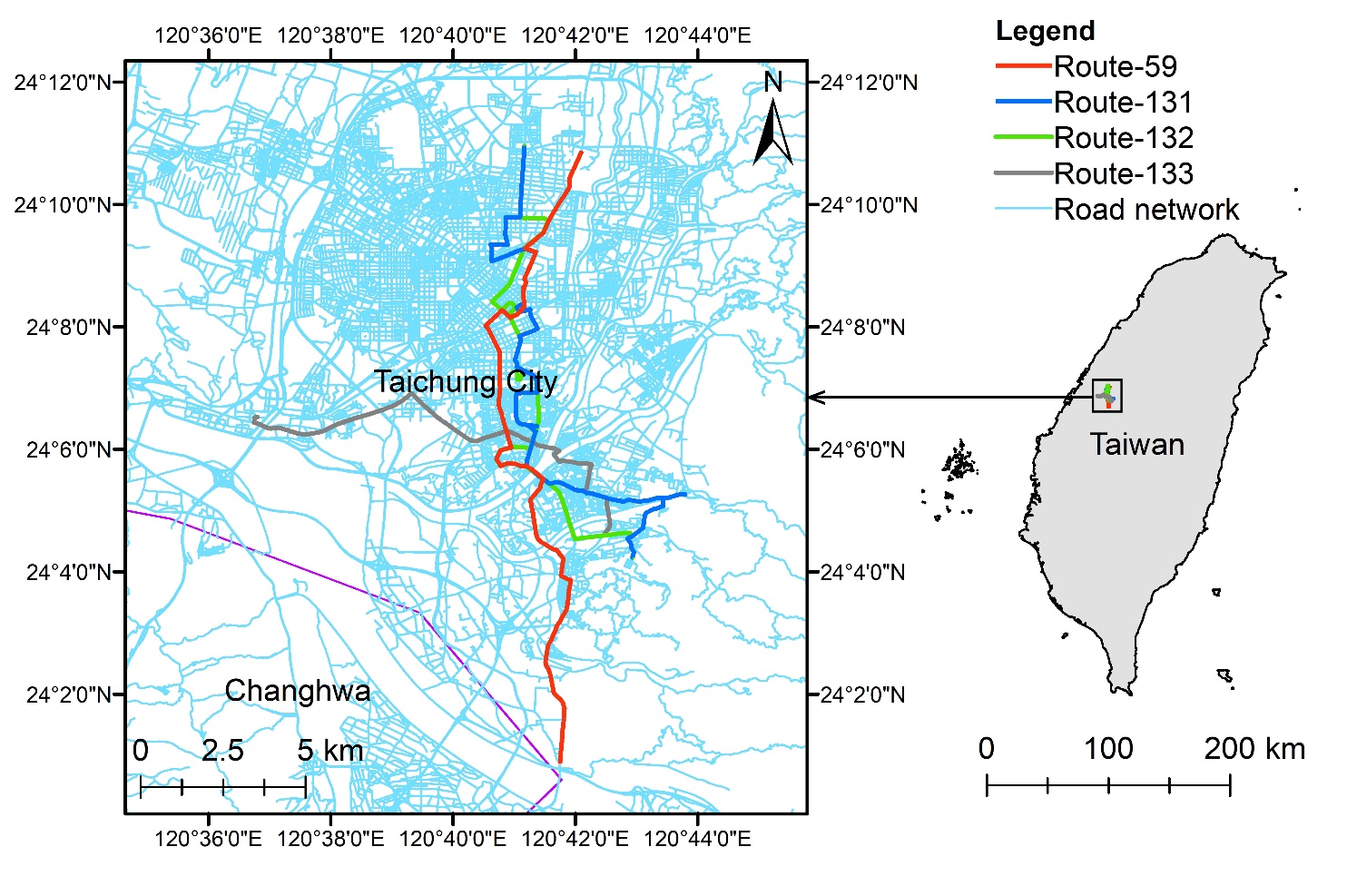


**Fig. S1** Map of the study area showing the sample bus routes


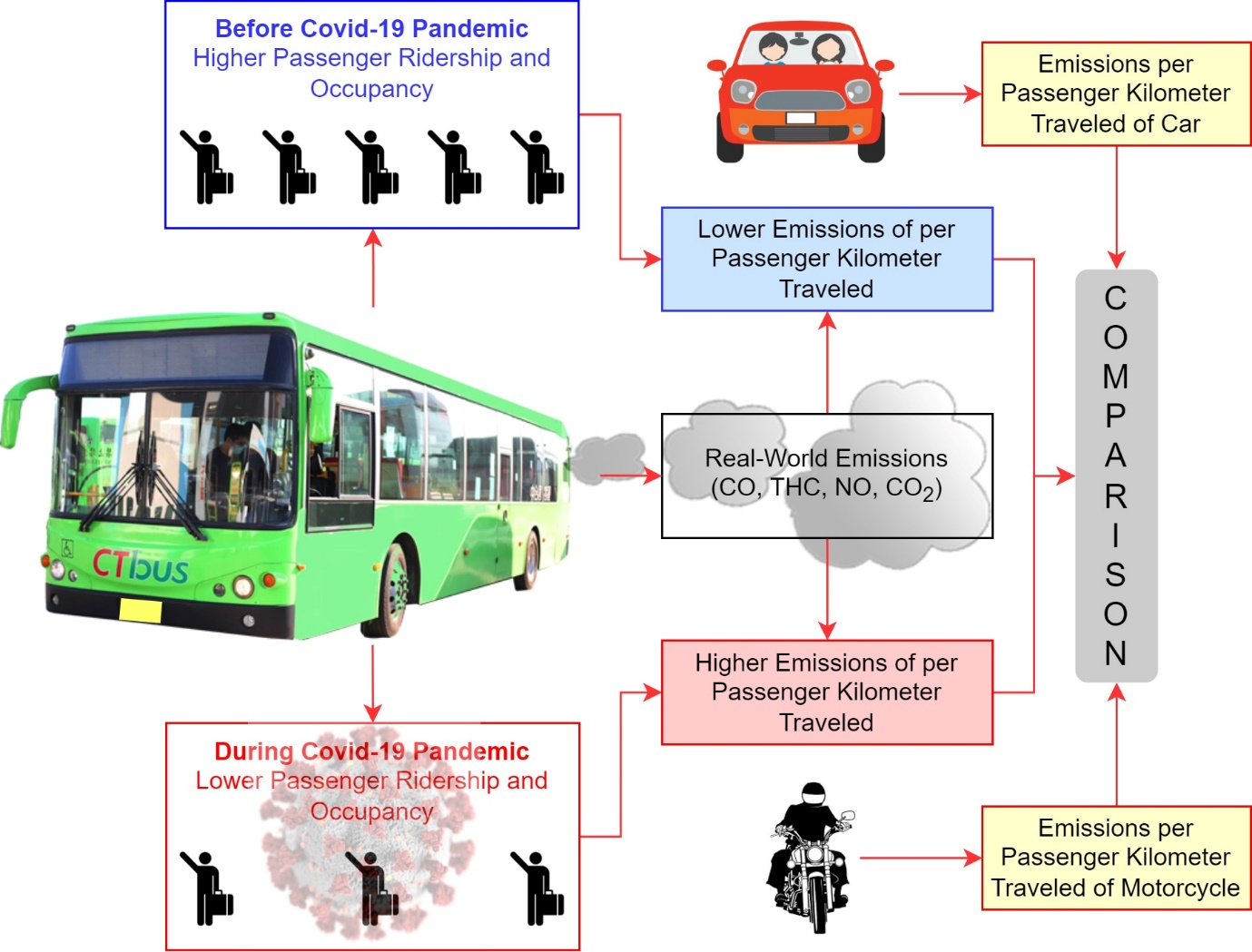


**Fig. S2** Schematic of the study design

**References**

1. Tsai JH, Yao YC, Huang PH, Chiang HL. Criteria pollutants and volatile organic compounds emitted from motorcycle exhaust under various regulation phases. Aerosol Air Qual Res. 2017;17:1214–23.
2. Tsai JH, Yao YC, Huang PH, Chiang HL. Fuel economy and volatile organic compound exhaust emission for motorcycles with various running mileages. Aerosol Air Qual Res. 2018;18:3056–67.
3. Yao YC, Tsai JH, Wang IT. Emissions of gaseous pollutant from motorcycle powered by ethanol-gasoline blend. Appl Energ. 2013;102:93–100.
4. Tsai JH, Huang PH, Chiang HL. Characteristics of volatile organic compounds from motorcycle exhaust emission during real-world driving. Atmos Environ. 2014;99:215–26.
5. Yang HH, Dhital NB, Cheruiyot NK, Wang LC, Wang SX. Effects of road grade on real-world tailpipe emissions of regulated gaseous pollutants and volatile organic compounds for a Euro 5 motorcycle. Atmos Pollut Res. 2021;12:101167.
6. MOTC. Motor Vehicle Registrations. Taipei: Ministry of Transportation and Communications; 2021 [in Chinese]. https://stat.motc.gov.tw/mocdb/stmain.jsp?sys=220&ym=8101&ymt=11106&kind=21&type=1&funid=b330101&cycle=41&outmode=0&compmode=0&outkind=1&fldspc=0,3,7,1,10,1,16,1,21,2,31,2,&rdm=yfeYbthy. Accessed 20 Jan 2022.
